# The social pragmatics of address in heritage Spanish: a virtual reality study

**DOI:** 10.3389/fpsyg.2026.1719331

**Published:** 2026-02-24

**Authors:** Abel Cruz

**Affiliations:** Department of Modern Languages and Literatures, Santa Clara University, Santa Clara, CA, United States

**Keywords:** heritage bilingualism, morphosyntax, pragmatics, production data, virtual reality

## Abstract

This study employed virtual reality to study heritage bilinguals’ pragmatic and morphosyntactic use of pronouns of address in heritage Spanish, namely the *tú/usted* paradigm. Forty-four heritage speakers experienced eight virtual scenarios embedding an array of social factors, such as gender and social rank of the addressee. This experimental design elicited a corpus of 21,882 words, which includes 753 instances of second-person address distributed into the *tú* (50.60%) and *usted* (49.40%) pronouns. Importantly, participants produced the *tú/usted* paradigm across all the expected syntactic environments, indicating that the feature geometry of this linguistic paradigm does not constrain its everyday use in heritage Spanish. A mixed-effects analysis further revealed that participants were more likely to express formality with female and unknown interlocutors. Since the majority of the participants are female (88.09%), a separate analysis confirmed the gender effect, which suggests that female speakers conveyed in-group solidarity through their pronoun usage in female-to-female interactions. Although participants produced the *tú/usted* paradigm across the target scenarios, they reported higher usage of *usted* (68.45%) in their perception data compared to their production data (49.40%), revealing a discrepancy between what speakers believe they do with language and how they actually behave in context. By studying the nuances of address in virtual worlds, the present study contributes to our current understanding of pragmatics in heritage bilingualism.

## 1 Introduction

When interacting with others, speakers must employ some linguistic form or sign to engage in a communicative exchange with their interlocutor. These linguistic forms are conceptualized as address systems in linguistics research, and pronouns are the most common forms of address cross-linguistically. The use of a particular form of address signals how the speaker wishes to be perceived by her interlocutor, e.g., the desire to be approved of or liked. Thus, in languages with formality distinctions in the pronominal system (e.g., the T/V dichotomy in Brown and Gilman, 1960), the speaker must assess the relative power of addressee over speaker, the social distance between speaker and addressee, and the degree to which a particular pronoun of address is rated as an imposition in the target culture (Brown and Levinson, 1978). Failure to make such assessment in a spontaneous interaction can expose the speaker to negative judgments in a speech community, e.g., being perceived as disrespectful or annoying, namely because formality is often correlated with age and unfamiliarity.

The everyday use of address systems involves both structural agreement and social pragmatic norms. As such, they serve as an ideal testing ground for exploring heritage speakers’ morphosyntactic and pragmatic knowledge. For instance, Spanish has a binary system for second-person singular address, namely the pronouns *tú* and *usted* (henceforth, the T/U paradigm). Following the feature geometry in Harley and Ritter (2002), the *tú* and *usted* pronouns are presumably specified as PARTICIPANT in the speech act with ‘addressee’ as their value, as illustrated in (1). However, this pronominal paradigm exhibits different verb agreement: the *tú*-pronoun inflects for second-person singular (e.g., *Tú tienes*), whereas the *usted*-pronoun exhibits third-person singular verb agreement (e.g., *Usted tiene*). In other words, *usted* requires activation of the ‘addressee’ node dependent on PARTICIPANT in (1), but the notionally perceived person of this node (2nd person) does not condition verb agreement.^1^

(1) Feature geometry of the T/U paradigm in Spanish. 
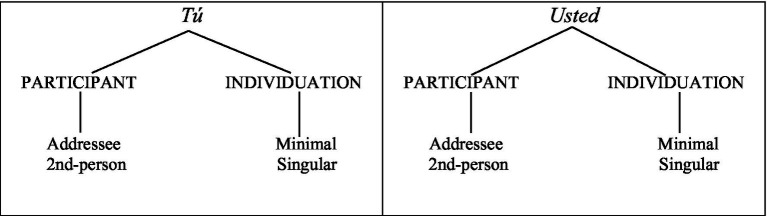


The T/U paradigm is then an addressee-sensitive variable subject to pragmatic norms in a speech community. Naturally, norms of address, which can be understood as conditions of (in)appropriate application of a form of address, emerge from our social relations with others, including parents and teachers. Child language studies indicate that by two to 3 years of age, children have acquired a robust understanding of social norms and their conditions of (in)appropriate application (Rakoczy and Tomasello, 2009; Schmidt and Rakoczy, 2023). In particular, children show awareness of social factors such a person’s social rank in society and solidarity for others early in life, and they consequently mitigate their speech (e.g., use politeness) to assess their social relations with others (Ervin-Tripp et al., 1990; Emihovich, 2008; Ryckyebusch and Marcos, 2004). In heritage bilingualism, however, heritage speakers learn their heritage language at home, but they are educated in the language of the broader speech community, English in the U. S. context (Valdés, 2005). Consequently, in most cases, English becomes their dominant language, and their heritage language is relegated to home usage. This learning trajectory limits heritage speakers to pragmatic norms of informal language use, while they learn other registers of language socialization through schooling in English (Carrira and Kagan, 2011; He, 2011; Polinsky, 2018).

While this learning trajectory suggests lack of cultural reference in the heritage language, there is an emerging body of literature showing that pragmatic competence in heritage bilingualism is sensitive to speakers’ heritage identity and bilingual experience (Cruz, 2024; Dubinina, 2021; Escalante and Quan, 2023; Pinto, 2018; Taguchi et al., 2017; Wolski-Moskoff, 2018; Xiao-Desai, 2019). Here, I understand pragmatic competence as “the speaker’s knowledge and use of rules of appropriateness and politeness” (Koike, 1989, p. 279). In the case of social relations, for example, heritage bilinguals employ pronouns of address to position themselves as members of a speech community, or to construct social identity (Liebscher et al., 2010; Warditz, 2025). Clearly, more studies are needed to have a better understanding of heritage speakers’ speech styles and pragmatic norms in their heritage languages (Polinsky, 2018). The present study aims to contribute to this line of research by examining Spanish heritage speakers’ use of pronouns of address in simulated interactions mimicking real-world scenarios.

The learning trajectory of heritage languages (e.g., learning contexts of unbalanced input) may also have structural consequences for heritage grammars (Flores et al., 2020; Montrul, 2023; Polinsky and Scontras, 2019, among others). In particular, grammatical phenomena such as agreement morphology have been reported to suffer in heritage Spanish in the U. S. context (Silva-Corvalán, 1994; Montrul, 2002; Polinsky, 2018), though studies in other languages in non-U. S. contexts report agreement patterns that closely resemble those of homeland speakers (Łyskawa and Nagy, 2020). While previous studies indicate that person agreement is mostly a stable feature in heritage grammars (Benmanmoun et al., 2013), I am not aware of any studies that have empirically investigated heritage speakers’ morphosyntactic knowledge of the T/U paradigm. As mentioned before, this linguistic paradigm exhibits a discourse-driven morphosyntactic mismatch that may cause morphological ambiguity for the heritage language learner, who may favor *tú* for second-person singular addressing because of its meaning-to-form mapping for verb agreement.^2^

The aim of the present study is then two-fold: to examine whether/how the feature geometry of the T/U paradigm constrains heritage speakers’ choice of pronoun of address in spontaneous speech and the social factors (pragmatic norms) that may inform their choice. The social factors governing heritage speakers’ use of pronouns of address in social contexts have received much attention in heritage bilingualism (Fernández-Mallat and Newman, 2021; Jaramillo, 1988, 1996; Sigüenza-Ortiz, 1996; Warditz, 2025). Yet, these studies rely mainly on questionnaires or translation tasks for data elicitation. While these methodologies provide valuable data, they are challenged with ecological validity. For example, in a traditional discourse completion task, participants are asked to imagine being in different scenarios where they would interact with others, creating an artificial spatial divide between participant and addressee (Peeters, 2019).

Noting this limitation, the present study employs Virtual Reality (VR) to elicit production data from heritage speakers’ use of the T/U paradigm. This new technology combines high ecological validity with high experimental control (Peeters, 2019; Titus et al., 2024). In particular, virtual reality allows for creating dynamic and interactive virtual worlds that generate a sense of presence, that is, the subjective experience of being in one place or environment when one is physically situated in another location (Witmer and Singer, 1998). VR is a compelling methodology for the study of address systems because it gives participants the opportunity to assess speaker-addressee relations in real time. The virtual scenarios created for the present study were carefully designed to reflect the social factors that have been previously reported to influence heritage speakers’ choice of pronoun in self-reported data. Importantly, production data is crucial to delimit whether preference for one particular pronoun over the other in a binary system is motivated by conventional norms of address in a given heritage community or by the feature geometry of the address system in the heritage language.

## 2 Literature review

### 2.1 Pronouns of address in the Americas

Brown and Gilman’s (1960) classic model of pronoun usage is the predominant framework to study address systems cross-linguistically. According to these authors, the semantics of power and solidarity mediate pronoun usage in some European languages, including the Romance languages, and the T/V dichotomy originating from the pronominal system of Latin captures these social dimensions. In a power relation, then, the inferior gives deferential V and receives subordinating T. In a solidary relation, by contrast, power equals give and receive reciprocal T. Social factors such as age, social rank, and gender—which pertain to both the speaker and the addressee—condition the use of the T/V dichotomy in a given speech community. The T/U paradigm that concern us here has been mainly studied within the T/V model, whereby *usted* conveys deference and *tú* expresses solidarity.

Spanish–English bilinguals have different linguistic forms at their disposal for expressing deference ad solidarity. In American English, for example, the pronoun ‘you’ refers to many persons or one, but this pronoun does not encode the semantics of power and solidarity described above (Brown and Gilman, 1960). Instead, other forms of address, such as a person’s title followed by last name or a person’s nickname, are used to convey these social dimensions (Brown and Ford, 1961). In contrast to American English, the Spanish spoken in the Americas has a tripartite system for second-person singular address, namely, *tú*, *vos*, and *usted*, and only one plural counterpart, *ustedes* (Fontanella de Weinberg, 1999). Thus, Spanish-speaking regions in the Americas are classified into three main dialectal regions: *tuteo*, *voseo,* and *ustedeo* (Cepeda Ruiz, 2018; Fernández-Mallat and Barrero, 2023). *Tuteo* and *voseo* refer to the use of the second-person singular pronouns *tú* and *vos*, respectively. These pronouns exhibit different verb morphology (e.g., *tú eres* vs. *vos sos* for present tense), but their object pronoun is always the same, *te*; their related possessives are *tu* and *tuyo* for both *tú* and *vos*. *Ustedeo* refers to the use of second-person singular pronoun *usted*, which derived from the reverential phrase *vuestra merced* ‘your grace’ (Moreno, 2002). As mentioned before, the pronoun *usted* exhibits a discourse-driven morphosyntactic mismatch for verb agreement, and this mismatch is also relevant for agreement with (in)direct object pronouns and possessives.

Contextual features and social factors play a crucial role in pronoun usage cross-linguistically, and those governing the T/U paradigm across the Americas are well documented in the literature (see Fernández-Mallat and Barrero, 2023 for a brief overview). For sake of space, here I limit the discussion to the social factors governing the T/U paradigm in Mexican Spanish. In a corpus study of Mexico City, Lastra (1972) found that speakers employed the pronoun *tú* almost exclusively in the familial domain, but they also extended it to people who have high social ranks in the Mexican society, such as priests and teachers. In another study from Mexico City, Schwenter (1993) found an overwhelming use of *tú*, while *usted* was primarily used to address strangers, and regardless of their social rank or gender. In a more recent study, Cepeda Ruiz (2018) reported that *tú* is relatively more frequent than *usted* in present-day Mexico City, whereby younger speakers preferred the *tú* form but also reported using *usted* with older people. Interestingly, Cepeda Ruiz found that women, compared to men, preferred using the *tú* form because it is more ‘respectful’ and ‘elegant’ than *usted*.

In short, the Mexican society applies informal (solidarity) language use in the familiar domain, but social factors such as age, social rank, and unfamiliarity still motivate the use of the T/U paradigm in this speech community. Although the present study does not seek to make a comparison of pronoun usage to a baseline speech community (see Rothman et al., 2023 for arguments against the monolingual comparative normativity in bilingualism), most of the existing studies on pronoun usage in heritage communities in the U.S. context include heritage speakers of Mexican descent, as summarized in the next section. In fact, the majority (78.58%) of the participants in the present study have a parent born and raised in Mexico, while only six participants have a parent born in El Salvador (11.90%) or Guatemala (2.38%); the rest of the participants have a parent born in the U. S. but of Mexican descent (7.14%). While five participants were presumably exposed to Salvadoran Spanish, where the pronoun *vos* is often used instead of *tú*, none of the participants of Salvadoran heritage produced this pronoun in the present study. Previous studies show that Salvadorans in the U. S. accommodate their pronoun usage to that of the Mexican-American community (Rivera-Mills, 2011); this may be particularly relevant in California where Mexican Spanish is predominant. Thus, it is fair to assume that the target heritage population was primarily exposed to the T/U paradigm in their heritage communities.

### 2.2 Norms of address in heritage bilingualism

Norms of address are part of heritage speakers’ pragmatic competence, or “the speaker’s knowledge and use of rules of appropriateness and politeness” (Koike, 1989, p. 279). Importantly, pragmatic competence in heritage bilingualism must include speakers’ heritage identity and bilingual experience (Dubinina, 2021; Escalante and Quan, 2023; Pinto, 2018; Taguchi et al., 2017; Wolski-Moskoff, 2018; Xiao-Desai, 2019). While studies on heritage pragmatics are scarce, the use of the *tú/usted* paradigm in heritage Spanish is well documented in the literature, though current studies stem primarily from self-reported pronoun usage elicited via questionnaires. For example, Brown (1971) surveyed 59 Spanish–English bilinguals from Tucson, AZ on their use of the T/U paradigm with their parents. Interestingly, more than twice as many participants across genders (63%) reported using *usted* with their parents. Similarly, Spanish–English bilinguals from Tomé, NM reported categorical usage of *usted* with their parents and, to a lesser extent, with co-workers regardless of their social rank (Jaramillo, 1988). Jaramillo’s study further showed that older bilinguals (50 + years) gave and received *usted* more frequently than their younger counterparts, which Jaramillo interpreted as reflecting the sociological differences in the Tomé community, whereby participants who grew up after World War II were innovating toward greater use of *tú* (e.g., more egalitarian norms).

In fact, more recent studies report greater use of *tú* across Spanish heritage communities in the U. S. context. In a follow-up study, for instance, Jaramillo (1996) surveyed 108 Spanish–English bilinguals from Tucson, AZ, divided into three age groups: 17–30, 31–50, and +51 years-old. The study controlled for participants’ age, gender, and domain of interaction (e.g., nuclear, extended or ceremonial family, *compadrazgo*) as potential factors informing speakers’ choice of pronoun. Participants across age groups and gender reported using *tú* categorically with nuclear family but less so with their extended family, and least frequently with ceremonial family. Moreover, in a study of Spanish–English bilinguals from Pico Rivera, CA, Sigüenza-Ortiz (1996) found categorical use of *tú* in the familial domain, but most of the participants in that study were observed using *usted* at church.

The studies discussed so far provide self-reported data, but more recent studies have applied other methodologies to study heritage speakers’ choice of pronoun of address. First, in a qualitative study, Gutiérrez-Rivas (2010) investigated how second- and third-generation Cuban Americans from Miami use the T/U paradigm when making requests in Spanish; second-generation included participants who were born in Cuba but arrived to the U. S. before age 12. All participants (*n* = 20) engaged in three role-plays involving different social relations: a neighbor-to-neighbor relation, an employer-to-employee relation, and a friend-to-friend relation. After completing the role-plays, participants were further asked which of the *tú*-*usted* pronouns they would use with strangers. Although both groups used the *usted* pronoun in their requests across the target role-plays, they produced greater usage of *tú*, especially the third-generation group; unfortunately, the author did not cross-tabulate the data by scenario, so it is unclear whether the social factors involved in the role-plays influenced participants’ use of the T/U paradigm. As for their reported usage of the T/U paradigm, 60% of the participants reported using *usted* when addressing a stranger, a usage pattern that suggests a discrepancy between produced and perceived pronoun usage by the target population.

More recently, Fernández-Mallat and Newman (2021) developed an innovative translation task in which participants viewed a picture, read an accompanying statement in English, and translated it into Spanish. The scenarios involved in this translation task controlled for social factors such as age, gender, social status, and distance, among other contextual and linguistic factors such as setting and speech act. The study included a sample of Spanish–English bilinguals (*n* = 149) who lived their childhood in New York City (65%) or in Latin America (35%). In general, these bilinguals used *tú* overwhelmingly (83.19%) over *usted* (15.78%) in their translations; in fact, 33 participants applied categorical use of *tú*. Their statistical analysis further revealed that participants were more likely to use *usted* with strangers and people who hold high social ranks, such as professors and employers. Though an innovative task, it is important to note that a translation task requires metacognition.

To summarize, heritage speakers of Spanish across speech communities in the U. S.-context show a strong preference for the *tú* over the *usted* pronoun, especially in studies conducted in the 1990s and thereafter. In particular, bilinguals reported using *tú* almost categorically within the nuclear family, while *usted* is more common in extended and ceremonial family relations, with unfamiliar interlocutors, and in church settings. Furthermore, these studies demonstrate that social factors such as age, social rank, unfamiliarity, and setting inform heritage speakers’ use of the T/U paradigm. Finally, Gutiérrez-Rivas (2010) study indicates that heritage speakers’ production of the T/U paradigm may not necessarily match their perceptions, but it is important to note that her study included only one question about speakers’ perceived use of the T/U paradigm. However, existing studies rely primarily on self-reported or written data and rarely incorporate objective measures of language proficiency to assess its potential role in pronoun usage. In addition, most studies report only relative frequencies of pronoun usage. Statistical analyses appropriate for binary variables are crucial to capture heritage speakers’ pronoun usage.

As mentioned before, speakers’ choice of pronoun can be manifested through subject pronouns, object pronouns, possessives, and through verb morphology. Thus, production data is crucial to delimit whether the reported preference for *tú* in heritage Spanish in U. S.-context is motivated by conventional norms of address in heritage bilingualism (e.g., use of language informal practices) or by the feature geometry of the T/U paradigm in the heritage language (e.g., where the morphosyntax of the grammar constrains the speaker’s realization of the T/U paradigm in favor of one-to-one mapping). Building on the observation that heritage speakers invoke social factors such as age, social rank, and social distance in their pronoun usage, the present study develops an interactive experimental design to investigate how these social factors influence heritage speakers’ use of the T/U paradigm in simulated, real-world interactions, thereby addressing some of the limitations noted above. In particular, the present methodology makes it possible to control for social factors from the addressee’s perspective.

## 3 Materials and methods

### 3.1 Research questions and predictions

*RQ1*: Does the feature geometry of the T/U paradigm constrain heritage speakers’ choice of pronoun in production data?

Prediction: Drawing on previous findings indicating that agreement morphology suffers in heritage Spanish (Silva-Corvalán, 1994; Montrul, 2002), we predict that the feature geometry of the T/U paradigm constrains heritage speakers’ pronoun choice in favor of *tú* because of its meaning-to-form mapping.

*RQ2*: What social factors inform heritage speakers’ use of the *tú/usted* paradigm in simulated interactions mimicking real-world scenarios?

Prediction: Given the predominantly informal context of heritage language development and lack of formal instruction in the heritage language (Carrira and Kagan, 2011), addressee’s age, perceived social rank or the social distance between speaker and addressee will not influence heritage speakers’ use of the pronoun *usted*, which conveys deference in the T/V dichotomy.

*RQ3*: Does heritage speakers’ production of the T/U paradigm match their perceptions about the use of this linguistic paradigm?

Prediction: Drawing on Gutiérrez-Rivas (2010) preliminary findings of a heritage community in Miami, heritage speakers are expected to report higher perceived use of *usted*, while producing limited use of this pronoun in their production data.

### 3.2 Participants

Forty-four heritage speakers of Spanish (5 males) between the ages of 19 and 26 (*M* = 20.40, SD = 1.39) participated in this study. All participants were recruited in a university setting in Northern California. Two participants were excluded due to late exposure to English (age 13) or malfunction of the recording. The final sample included forty-two participants (5 males) between the ages of 19 and 26 (*M* = 20.40, SD = 1.41). Thirty-three of the participants were born or raised in California, and two participants were born outside of the U. S. but arrived to California at ages eight and 10. The rest of the participants (*n* = 7) were born and raised in other states across the U. S., including Colorado (*n* = 2), Idaho (*n* = 2), Washington (*n* = 1), Arizona (*n* = 1), and New York (*n* = 1). All participants lived in Northern California at the time of the study. Participants completed a modified version of the Language and Social Background Questionnaire (LSBQ) (Anderson et al., 2018), which collected participants’ demographics, language background information, and self-reported proficiency in Spanish and English. Proficiency in Spanish was further assessed with the Spanish Elicited Imitation Task (EIT) validated in Bowden (2016), which is a slightly modified version of a shortcut measure of language proficiency developed by Ortega et al. (2002).

Participants’ language profile information is summarized in Table 1. The majority of the participants (95.24%) reported exposure to Spanish from birth, while one participant reported exposure to Spanish at age 3 and another participant at age 11 (*M* = 0.33, SD = 1.74). Exposure to English ranged from birth to age 10 (*M* = 3.45 years, SD = 2.60). As for language proficiency, participants reported high proficiency in both Spanish and English, and in both oral communication and reading and writing. The EIT proficiency measure confirmed participants’ high proficiency in Spanish with a mean of 106.47 (SD = 11.59); for example, in Bowden’s (2016) validation of the EIT version used in this study, a mean of 109.3 (SD = 8.2) represented her ‘very advanced’ group of Spanish language learners. Finally, participants reported, on average, greater Spanish use with family members (68.88%) than English (39.19%), whereas with friends they reported greater English use (87.23%) than Spanish (30.38%) on a weekly basis. In short, the target heritage population represents early Spanish–English bilinguals who were immersed in a bilingual experience either from birth or as early sequential bilinguals and who use their two languages in diverse contexts and with different groups of people (e.g., Ortega, 2020).

**Table 1:** Participants’ characteristics and bilingual profile information.

Participants’ characteristics	*M* (SD)	min–max
Age at testing (years)	20.40 (1.41)	19–26
Onset age of exposure to Spanish	0.33 (1.74)	0–11
Onset age of exposure to English	3.45 (2.60)	0–10
Self-rated Spanish proficiency in		
Reading	6.07 (0.86)	4–7
Writing	5.6 (1.05)	3–7
Speaking	5.90 (0.98)	4–7
Listening	6.54 (0.63)	5–7
Self-rated English proficiency in		
Reading	6.57 (0.57)	5–7
Writing	6.5 (0.77)	4–7
Speaking	6.57 (0.63)	5–7
Listening	6.66 (0.61)	5–7
Spanish EIT Scores	106.47 (11.59)	71–120
Spanish language use in percentage (%) per week		
With family	68.88 (30.53)	10–100
At work	20.71 (19.74)	0–90
With friends	30.38 (28.33)	0–90
English language use in percentage (%) per week		
With family	39.19 (30.01)	0–100
At work	86.70 (14.47)	40–100
With friends	87.23 (14.34)	50–100

Self-ratings are from 1 = not proficient at all to 7 = highly proficient. Spanish EIT maximum total score is 120.

Parents’ birth place is also relevant for the present study. In particular, the majority (78.58%) of the participants have a parent who was born and raised in Mexico. The rest of the participants have a parent born and raised in El Salvador (11.90%), the U. S. (7.14%), or Guatemala (2.38%). Thus, the majority of these heritage bilinguals were presumably exposed to Mexican Spanish.

### 3.3 Materials

The present study employed virtual reality to elicit production data from heritage speakers’ use of the T/U paradigm. This new technology allows for creating virtual worlds that are as dynamic, interactive, and rich as the real world. The VR experience involved a head-mounted display that gave participants a 360-degree view of the target virtual scenarios, creating a high sense of presence in the virtual environment (Witmer and Singer, 1998). The program was created in the software program Unity 5. Eight different scenarios framed within a study abroad journey were created (see Supplementary Table S1 for summary of scenarios). The scenarios were carefully designed to control for the interlocutor/addressee’s (virtual agent) age, gender, social rank, and social distance in relation to the speaker/participant. All virtual agents were matched with human voices of their respective age and gender. The setting of the virtual environment also contributed to the expected social relations between participant and interlocutor. Participants also perceived motion from other agents participating in the 3D environment, which aimed to enhance presence in the VR experience. The VR game was 13 min long. The files to use the VR game for future studies can be accessed in the author’s Open Science Framework (OSF).^3^

In the interactions, the participant played the role of an international college student participating in a study abroad program in the School of Economics at the University of Mexico in Mexico City. As described before, the ages of the participants ranged from 19 to 26 (*M* = 20.40). Therefore, the age and gender factors (equally divided) are in relation to the perceived age and gender of the addressee, not the participant. The ‘power’ factor (equally divided) refers to the addressee’s assigned social rank, e.g., a person’s professional title or family hierarchy. Finally, the ‘distance’ factor refers to whether the addressee is known or unknown to the participant; in the next section, I discuss how these social factors were coded in the analysis. Although it would be desirable to have more scenarios, the scenarios designed for the present study were successful in eliciting a large corpus of production data from a large number of participants.^4^

Participants also completed an Immersiveness Questionnaire (IQ) and a Language Awareness Questionnaire (LAQ). In the IQ, which used a five-point scale (1 = not at all immersed, 5 = very immersed), participants rated how immersed they felt in each of the scenarios they experienced in VR. In the LAQ, participants further indicated which of the *tú* and *usted* pronouns they would use for each of the scenarios they experienced in VR and to justify their selection. In the LAQ, participants answered two open-ended questions: (a) In general, in what situation would you say you use the pronoun *tú* when interacting with others in Spanish? and (b) In general, in what situation would you say you use the pronoun *usted* when interacting with others in Spanish? These questions probed participants’ understanding of the social factors that govern the use of the T/U paradigm. Finally, as part of the LAQ, participants indicated which pronoun they use when addressing their parents, siblings, aunts and uncles, grandparents, and their religious leader, if applicable.

### 3.4 Procedure

This study was approved by the author’s Institutional Review Board. Participants provided written consent before starting the VR experience, which took place in a quiet room. Once consent form was obtained, participants were told that they would be wearing a headset to immerse themselves in a virtual reality experience that simulated a study abroad journey to the University of Mexico in Mexico City. Participants were further told that they would encounter different scenarios throughout their virtual experience in which they would engage in a conversation in Spanish with virtual agents who would ask them questions about their journey to Mexico.

The immersive experience started with written instructions in English, which indicated that participants were participating in a study abroad program with the School of Economics at the University of Mexico and instructed participants to use Spanish in their interactions and to be as natural as possible in their conversations. Before engaging in a conversation, participants read a short prompt in the VR environment that briefly explained the upcoming scenario and the kind of information they were supposed to request or share with their addressee. The virtual agents initiated the conversation with an opening phrase in Spanish (e.g., *¡Hola! ¿Estás bien? ¿Te puedo ayudar en algo?* ‘Hi, are you okay? Can I help you?’), which in turn prompted the participant to respond in Spanish to the agent’s question. Each scenario was timed according to the nature of the expected response, and the program continued to the next scenario after the allotted time expired (the interested reader can watch the video of the VR game in the author’s OSF: https://osf.io/trcuh/).^5^ The interactions were audio recorded and transcribed using Sonix. The author carefully edited the transcriptions and annotated the data for analysis.

After the immersive experience, participants completed the Immersiveness Questionnaire, the Language Awareness Questionnaire, and the Language Background Questionnaire via Qualtrics. Finally, participants took the Spanish Elicited Imitation Task (EIT). Altogether, the study took approximately 50–60 min to complete. Participants who were enrolled in a Spanish language class received extra credit for their participation.

### 3.5 Analysis

The data analysis was carried out using mixed-effects logistic regression, performed with the lme4 package (Baayen et al., 2008; Bates et al., 2015) in the statistical software R (R Core Team, 2021). In particular, I fit a mixed-effects model testing participants’ choice of pronoun, *tú* or *usted*, a binary dependent variable. The social factors potentially informing participants’ choice of pronoun were the following:

1. Age, binary factor referring to the agents’ perceived age, equally divided into *same* age as participant and *older* than participant;
2. Gender, binary factor referring to the agents’ perceived gender, equally divided into *male* and *female*;
3. Power, binary factor referring to the agents’ perceived social rank, equally divided into *present* and *absent*;
4. Distance, binary factor referring to the social distance between participant and addressee, *known* or *unknown*;
5. Linguistic category, categorical factor with *verb*, *object clitic pronoun* and *other* (lone pronoun, which includes instances of *tú* and *usted* without verb morphology, and possessives) as its levels;
6. Spanish proficiency as a continuous variable.

Unfortunately, participants’ gender was not included in the analysis because the majority of the participants (88.09%) are female, only 5 male participants. I should also note that three participants defaulted to *tú* pronoun use, but none defaulted to *usted* usage. Moreover, as a reviewer noted, one participant reported age 11 as the onset age of exposure to Spanish. Thus, I ran three models for the present analysis; one including all participants (Model-1), one excluding four participants (Model-2), and a third model (Model-3) for female-only participants.

This study concerns a binary dependent variable, and so I followed the recommendations in Gries (2021), being careful to follow the steps in Cruz and Suethanapornkul (2025). First, I applied the following transformations to the predictors: categorial predictors were sum-coded and Spanish proficiency was grand-mean centered and standardized. The first model (Model-1) included all 42 participants (5 males). In building Model-1, which included a total of 753 instances of the variable of analysis, I first added the item-level (item = scenario in this case) predictors (age, gender, power, distance, linguistic category) and participant-level predictor(s) (Spanish proficiency) as fixed effects and checked variance inflation factors (VIFs), predictors were not correlated (all VIFs = 1 or 2). Then, I included intra-level interactions for four item-level predictors (power, distance, age, gender), but the *gender:power* and *gender:distance* interactions were dropped because they were not identifiable by VIFs. Next, I incorporated random slopes into the model, Model-1. Initially, I included by-participant slopes for age, gender, power, distance, and linguistic category, as well as by-scenario/item slopes for Spanish proficiency.

However, this maximal model returned a singular fit, so I simplified the structure by retaining intercepts for participant and scenario, as well as by-participant random slopes for ‘gender’ and ‘distance,’ which were significant in the model. Lastly, I incorporated cross-level interactions into the model, but none of these interactions were significant, so they were removed (Likelihood-ratio tests (LRTs) confirmed their removal, *χ*^2^ (8) = 11.407, *p* = 0.179). The predictors ‘age’ and ‘linguistic category’ had large *p*-values, and were subsequently removed (LRTs = *χ*^2^ (3) = 1.05, *p* = 0.789). The final model from which I drew inferences for Model-1 (all participants) was significantly different from the null model (LRTs = *χ*^2^ (4) = 20.525, *p* < 0.001; AIC_final model_ = 615.89; AIC_null model_ = 628.42) with VIFs equal to 1; the value for *R*^2^_marginal_ was 0.28, and *R*^2^_conditional_ was 0.90. The model’s C score was 0.97, which is above the 0.80 threshold commonly used in linguistics (Gries, 2021).

Model-2, which excluded four participants for reasons explained above, included 38 participants (5 males), and a data set of 696 instances of the *tú/usted* paradigm, *tú* = 369 and *usted* = 327. Following the same model preparation steps for Model-1, Model-2 was simplified by retaining intercepts for participant and scenario, as well as by-participant random slopes for ‘gender’ and ‘distance,’ which were also significant in the output for Model-2. Similar to Model-1, none of the cross-level interactions were significant, so they were removed from the model [LRTs = *χ*^2^ (8) = 9.157, *p* = 0.329], as was age and category for having large *p*-values [LRTs = *χ*^2^ (3) = 1.048, *p* = 0.789]. The final model was significantly different from the null model [*χ*^2^ (4) = 24.08, *p* < 0.000; AIC_final model_ = 581.41; AIC_null model_ = 597.49], with all VIFs equal to 1; the value for *R*^2^_marginal_ was 0.39, and R^2^_conditional_ was 0.90. The model’s C score was 0.97.

The reader may recall that the majority of the participants (*n* = 37, or 88.09%) are female. Thus, I ran a third model (Model-3) that included only female participants. The final specification of Model-3 included random-effect components of participant and scenario, as well as by-participant random slopes for ‘gender’ and ‘distance’ because these factors were significant in the model. Similar to the other two models, none of the cross-level interactions were significant, so they were removed from the model [LRTs = *χ*^2^ (8) = 8.89, *p* = 0.351], as was “age” and “category” for having large *p*-values [LRTs = *χ*^2^ (3) = 1.39, *p* = 0.707]. The final model for Model-3 was significantly different from the null model [*χ*^2^ (4) = 20.16, *p* < 0.001; AIC_final model_ = 566.99; AIC_null model_ = 579.16], with all VIFs equal to 1 or 2; the value for *R*^2^_marginal_ was 0.33, and *R*^2^_conditional_ was 0.90. The model’s C score was 0.97. The data sets and r-scripts for this study are available at OSF: https://osf.io/trcuh/.

## 4 Results

The experimental design employed in this study was successful in eliciting a large amount of production data from the target population, for a corpus of 21,882 words. Moreover, participants rated their immersive experience as “somewhat immersed” or “moderately immersed” (range: 3.66–4.24, see Supplementary Table S2 for full data set). Before discussing the results, it is important to highlight participants’ understanding of the social dimensions that govern the use of the T/U paradigm in their everyday speech. In their responses to the open questions on the language awareness questionnaire about their general use of the *tú* and *usted* pronouns, participants mentioned concepts like friendship, family, equal power, similar age, and informal settings as key social dimensions informing their use of the *tú* pronoun, whereas power dynamics, unfamiliarity, older in age, titles, parents, grandparents, and formal settings influence their use of *usted*. These comments are comparable with the social dimensions attributed to the T/U paradigm in other heritage communities, and in Mexican society more generally.

As for the variable of analysis, there are 753 instances of second-person singular address in the target corpus, 381 (50.60%) of which represent the *tú* pronoun and 372 (49.40%) represent the *usted* pronoun. These tokens are manifested in verb agreement, clitic pronouns, possessives, and the *tú/usted* pronouns, as reported in Table 2. Importantly, there are only a few instances of unexpected agreement with possessive pronouns, but they were excluded from the final data set in Table 2.

**Table 2:** Frequencies of the *tú/usted* paradigm by syntactic environment.

Variable	Lone pronoun	Verb morphology	Clitic pronoun	Possessive pronoun	Total
TU	10	219	90	62	381
USTED	24	212	92	44	372
Total	34	431	182	106	753

The ‘lone pronoun’ category in Table 2 includes instances of *tú* and *usted* without verb morphology (e.g., *para ti* or *para usted* ‘for you’). For the ‘verb agreement’ category in Table 2, the target verb could cooccur with the *tú/usted* pronouns as the subject, but the majority (87.47%) occurred without an overt subject; low frequencies of overt subject expression are expected because Spanish is a pro-drop language. The ‘clitic pronoun’ category in Table 2 includes direct and indirect clitic pronouns, and the ‘possessive pronoun’ category includes singular and plural possessives.

Table 3 further breaks the distribution of the T/U paradigm across the target scenarios. Table 3 illustrates that participants produced both pronouns across the same scenario. That is, and contrary to what we would expect given the social relations involved in the target scenarios, none of the scenarios triggered categorical use of *tú* or *usted*. Therefore, a mixed-effects logistic regression analysis was carried out to examine the social factors that may influence participants’ choice of pronoun across scenarios. Table 4 reports the results of the best fitting model for the full data set, Model-1 in the analysis.

**Table 3:** Distribution of the *tú/usted* paradigm by target scenario in production.

Variable	V1	V2	V3	V4	V5	V6	V7	V8	Total
TU	51	42	8	12	128	13	37	90	381
USTED	46	42	31	44	15	63	46	85	372
Total	97	84	39	56	143	76	83	175	753

**Table 4:** Participants’ likelihood of expressing formality through the *usted* pronoun.

Parameters	Estimates	SE	95% CI	*z*	*p*
(Intercept)	0.56	0.66	[−0.94, 1.88]	0.85	0.394
Gender_male vs. female_	−2.72	0.87	[−4.43, −0.99]	−3.09	0.001**
Distance_known vs unknown_	−2.76	0.83	[−4.40, −1.13]	−3.31	0.001***
Power_present vs absent_	0.71	0.43	[−0.13, 1.56]	1.65	0.098
Proficiency_Spanish_	0.83	0.46	[−0.06, 1.74]	1.83	0.067
Random Effects	Variance	SD			
Intercept | subject	10.35	3.22			
Intercept | scenario	0.80	0.89			

The 95% CIs were approximated using the Wald method; *p < 0.05; **p < 0.01; ***p < 0.001.

As mentioned before, I used sum-coding for categorical predictors, meaning that the estimates in Table 4 represent the deviation of each level from the grand mean of the intercept. Table 4 shows a significant and negative effect for gender, that is, the perceived gender of the interlocutor (*β* = −2.72, *SE* = 0.87, *z* = −3.09, *p* < 0.001). This negative estimate means that participants were less formal with male interlocutors and, conversely, more likely to be more formal with female interlocutors through their use of *usted*. Predictions from the model further revealed that the proportion of formality with female interlocutors was 45.2%, compared to 0.36% with male interlocutors. In other words, participants were significantly more likely to express formality through the use of *usted* when addressing female interlocutors than when addressing male interlocutors.

The model reported in Table 4 also revealed a significant and negative effect for “distance” (*β* = −2.76, SE = 0.83, *z* = −3.31, *p* < 0.001), meaning that participants were significantly more likely to express formality with unknown interlocutors than with known interlocutors. In particular, the proportion of formality with unknown interlocutors was 47.6%, compared to 0.35% with known interlocutors. On the other hand, power, or the social rank assigned to the addressee, (*β* = 0.71, SE = 0.43, *z* = 1.65, *p* = 0.098) and proficiency in Spanish (*β* = 0.83, SE = 0.46, *z* = 1.83, *p* = 0.067) did not reach significance; the reader may also recall that none of the interactions between proficiency and scenario-level predictors was significant. That is, in the full data set of 42 participants (Model-1), proficiency in Spanish did not influence participants’ expression of formality. Finally, recall that the “age” and linguistic “category” factors were removed from the final model because they did not contribute in a meaningful way.

The reader may also call that three participants defaulted to *tú* pronoun usage, and one participant reported late exposure to Spanish (at age 11). Thus, I ran a second analysis (Model-2) that excluded these four participants, for a total of 38 participants (5 males). This second analysis included 696 instances of the *tú/usted* paradigm. In this analysis (Model-2), gender (*β* = −2.98, SE = 0.93, *z* = −3.20, *p* < 0.001) and distance (*β* = −3.06, SE = 0.88, *z* = −3.47, *p* < 0.000) were also significant. Unlike the analysis with all participants (Model-1), proficiency also reached significance at conventional *p* < 0.5 level in Model-2 (*β* = 0.92, SE = 0.43, *z* = 2.17, *p* < 0.03). In other words, higher proficiency increased the odds of using *usted* over *tú* in Model-2 (see Supplementary Table S3 for full results of Model-2). In the next section, I provide some insights on this particular finding.

Since the majority of the participants are female (88.09%), I ran a third analysis for female-only participants (Model-3). The female-only data set consists of 677 tokens of the T/U paradigm almost equally distributed: 339 instances of *tú* (50.07%) and 338 of *usted* (49.93%) Table 5 reports the results for the best fitting model.

**Table 5:** Female participants’ likelihood of expressing formality through the *usted* pronoun.

Parameters	Estimates	SE	95% CI	*z*	*p*
(Intercept)	0.43	0.66	[−0.87, 1.74]	065	0.518
Gender_male vs. female_	−2.65	0.90	[−4.42, −0.89]	−2.95	0.003**
Distance_known vs unknown_	−2.68	0.86	[−4.38, −0.99]	−3.09	0.001**
Power_present vs absent_	0.90	0.43	[0.06, 1.75]	2.11	0.034*
Proficiency_Spanish_	0.79	0.49	[−0.17, 1.77]	1.61	0.106
Random Effects	Variance	SD			
Intercept | subject	9.45	3.07			
Intercept | scenario	0.74	0.86			

The 95% CIs were approximated using the Wald method; *p < 0.05; **p < 0.01; ***p < 0.001.

In the female-only model (Model-3), gender (*β* = −2.65, SE = 0.90, *z* = −2.95, *p* < 0.003) and distance (*β* = −2.68, SE = 0.86, *z* = −3.09, *p* < 0.001) were significant. Unlike the model with all participants (Model-1) and the one with participant exclusion (Model-2), ‘power’ reached a level of significance (*β* = 0.90, SE = 0.43, *z* = 2.12, *p* < 0.03) in the female-only model reported in Table 5, but proficiency was not significant (*β* = 0.79, SE = 0.49, *z* = 1.61, *p = 0*.106).

**Figure 1:**
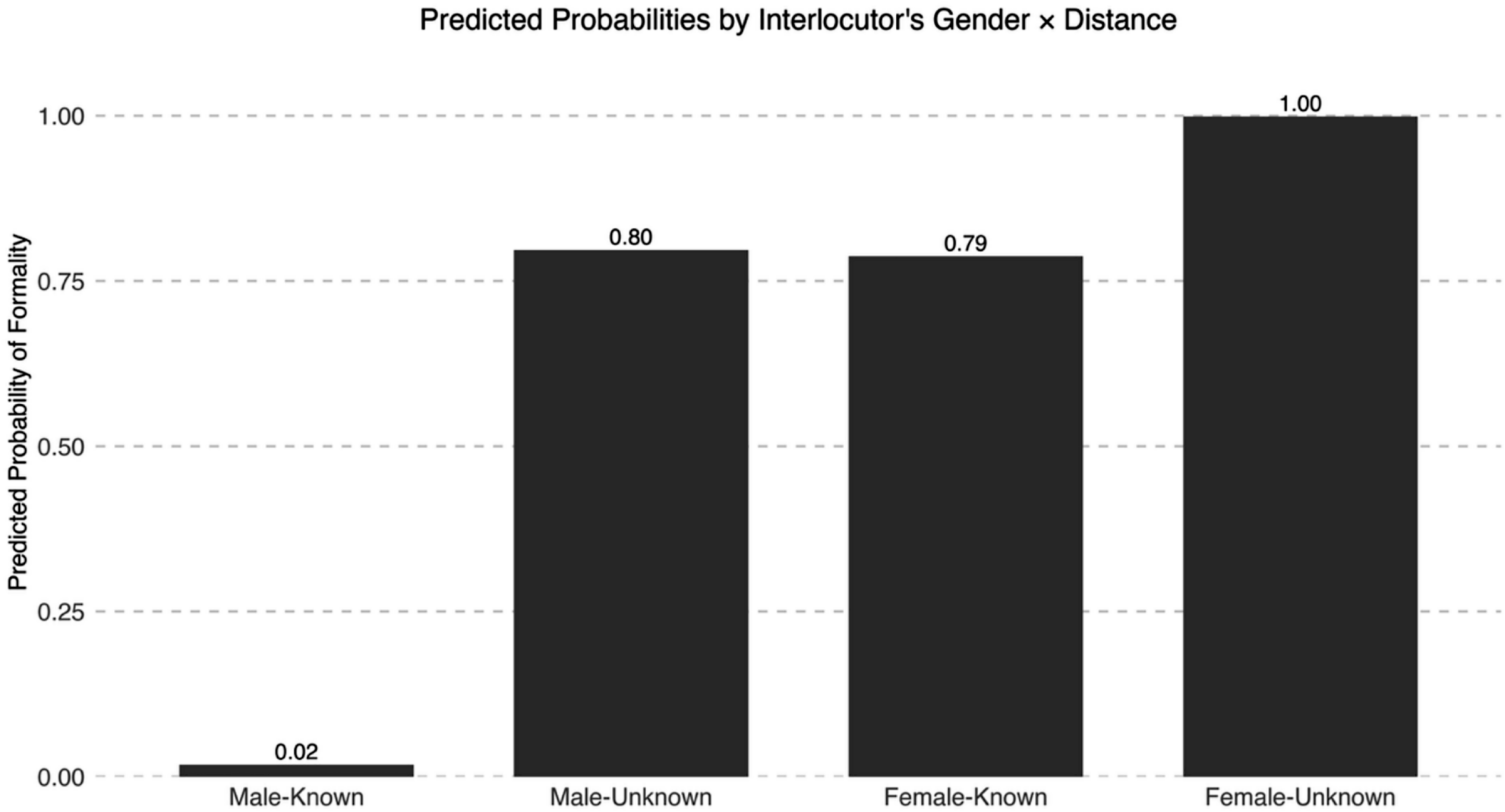
Predicted probabilities of expressing formality across gender and distance.

For the sake of illustration, Figure 1 reports the predicted probabilities of gender and distance for the female-only model, though this interaction was not significant. In Figure 1, we can see that female participants applied formality (e.g., use of *usted* pronoun) when addressing unknown male interlocutors. That is, they did not default to *tú* pronoun use with all male interlocutors, but the predicted probabilities indicate that they applied formality with unknown female interlocutors in a categorical fashion. In fact, female participants also applied formality with known female interlocutors; note that ‘known’ interlocutors included their professor and their grandmother, so power may be influencing their high probability illustrated in Figure 1.

**Figure 2:**
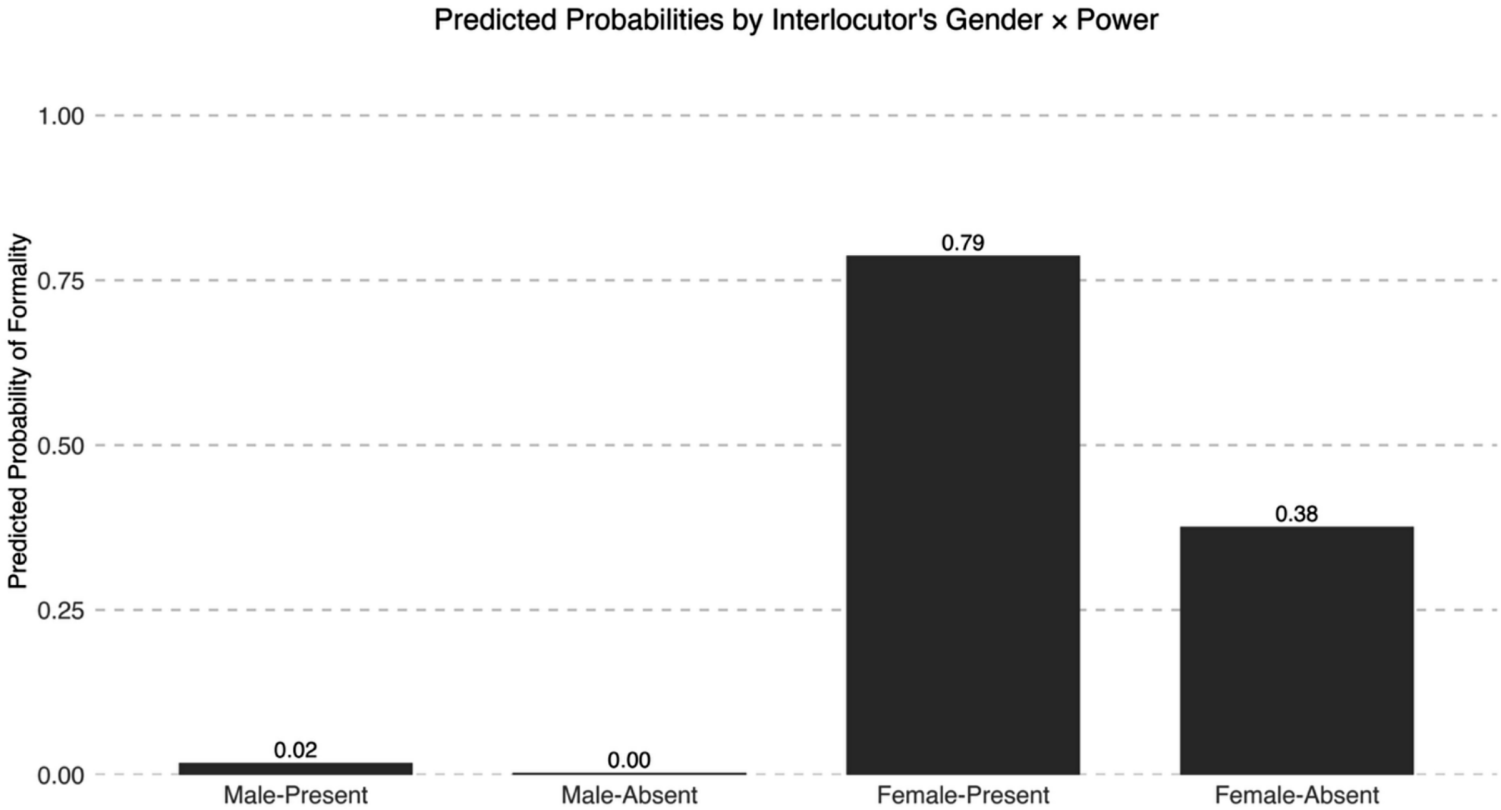
Predicted probabilities of expressing formality across gender and power.

‘Power,’ or social rank, also reached a level of significance in the female-only analysis reported in Table 5. Figure 2 shows that female participants were more likely to apply formality when addressing female interlocutors who hold high social ranks (e.g., a professor or university secretary), but not with male interlocutors who also hold power (e.g., potential employer). Similarly, participants also applied formality when addressing female interlocutors who do not hold high social ranks (e.g., a young waitress at a college cafeteria). In short, social factors such as the social distance between speaker and addressee and the perceived gender and social rank of the addressee informed female heritage speakers’ use of *usted* with female interlocutors. In the next section, I provide a possible explanation of these findings.

In the Language Awareness Questionnaire (LAQ), participants were further asked to indicate which of the *tú* and *usted* pronouns they would use for each of the interactions they experienced in VR and to briefly explain their selection. This questionnaire was included to explore whether participants’ production of the T/U paradigm would match their reported usage. Table 6 summarizes the percentages of participants (all participants) who reported either *tú* or *usted* across the target scenarios.

**Table 6:** Participants’ reported pronoun usage in percentages by target scenario.

Variable	V1	V2	V3	V4	V5	V6	V7	V8
TU	61.90%	7.14%	4.76%	57.14%	95.24%	0.00%	0.00%	26.19%
USTED	38.10%	92.86%	95.24%	42.86%	4.76%	100%	100%	73.81%

As we can see in Table 6, all participants reported using *usted* with their professor (V6) or potential employer (V7), and the majority reported *usted* usage with the university secretary (V3, 95.24%), a taxi driver (V2, 92.86%), and their grandmother (V8, 73.81%). These particular scenarios involve interlocutors older than the participant, and social rank for scenarios V3, V6, and V7, two factors (age and power) that were not significant in the analysis including all participants reported in Table 4. When asked to justify their choice of pronoun, participants stated that age, social rank, and professional setting informed their use of *usted*. On the other hand, the majority of the participants reported using *tú* with their male classmate (V5, 95.25%), and more than half of them reported *tú* usage with a stranger at the airport (V1, 61.90%) and with a female waitress at a college cafeteria (V4, 57.14%). In their justifications, participants stated that equal relations concerning age (same as participant), familiarity, and informality influenced their use of the *tú* pronoun in these scenarios (see Supplementary Table S4 for summary of full responses).

The results for scenario V4 are telling. This scenario involved a 20-year-old female waiter at a college cafeteria, but 42.86% of the participants reported using *usted* in this scenario. In fact, 78.57% of the production data in Table 3 represent *usted* pronoun usage for scenario V4. In their justifications, participants stated that they applied formality in such scenario because the waitress was providing a service for them, and they wanted to show politeness and respect for such disposition.

Finally, as part of the LAQ, all participants reported their pronoun usage with family members and, when applicable, with their religious leader. For these results, about 31% reported addressing their parents with *usted*, while all reported using *tú* with their siblings; and a majority uses *usted* with extended family: 59.52% with aunts and uncles, 71.43% with grandmothers, and 73.68% with grandfathers, with four participants selecting ‘NA’ for grandfather. Among those affiliated with the church (85.71%), all reported using *usted* with their religious leader. In the next section, I discuss how these findings inform address practices in the target heritage community.

## 5 Discussion

The present study employed virtual reality to investigate heritage bilinguals’ pragmatic and morphosyntactic use of 2nd-person singular pronouns of address in heritage Spanish, namely the *tú/usted* paradigm. Pronouns of address are of particular interest because social dimensions govern their everyday use, which are difficult to study in survey questionnaires or through the sociolinguistic interview in which participants remain consistent in their choice of pronoun. Thus, I reasoned that virtual reality gives participants the opportunity to assess social relations between speaker and addressee in real-time (Peeters, 2019; Wirtz et al., 2024). It is therefore fair to assume that the production data analyzed here, combined with speakers’ perception data, is representative of Spanish heritage speakers’ pragmatic and morphosyntactic knowledge of the T/U paradigm.

In this study, forty-two Spanish heritage speakers who lived in Northern California at the time of the study experienced eight virtual scenarios controlling for the interlocutor/addressee’s perceived age, gender, social rank, and social distance between speaker and addressee. This novel methodology elicited a total of 21,882 words. The results revealed that participants produced the T/U paradigm in an almost equal distribution: 50.60% of the *tú* pronoun and 49.40% of the *usted* pronoun. While previous studies reported a strong preference for the *tú* over the *usted* pronoun in other Spanish heritage communities in the U. S. (Brown, 1971; Fernández-Mallat and Newman, 2021; Jaramillo, 1996; Sigüenza-Ortiz, 1996), the majority of these studies reported perception rather than production data. However, I stressed that production data is needed to delimit whether this reported preference is promoted by the morphosyntactic mismatch of the T/U paradigm for verb agreement, which favors the use of *tú* because of its meaning-to-form mapping, or by conventional norms of address in a heritage community (e.g., informal language use).

Thus, RQ1 asked whether/how the feature geometry of the T/U paradigm in (1) constrains heritage speakers’ choice of pronoun in spontaneous speech. I predicted that participants would use *tú* more frequently than *usted* because of its meaning-to-form mapping for verb agreement. However, the results showed that the target population produced both pronouns across the syntactic environments relevant for 2^nd^-person singular address. In fact, the results indicate that participants produced the T/U paradigm in similar frequencies across the syntactic environments relevant for the target variable (see Table 2). Moreover, only a few instances of agreement mismatches were observed with possessive pronouns, but none with verb agreement or object clitic pronouns. Finally, of the 431 instances of 2^nd^-person singular address manifested in verb agreement, the majority (87.47%) occurred without an overt subject expression. While this usage pattern is expected given that Spanish is a pro-drop language, previous studies have reported that heritage bilinguals overuse overt pronouns in contexts that normally call for a null form (Montrul, 2002; Otheguy et al., 2007).

The present study then suggests that the category of ‘person’ is not a vulnerable feature in the grammar of the target heritage population, as demonstrated by participants’ agreement patterns of the *tú/usted* paradigm in spontaneous speech. This finding is consistent with the observation that person agreement is mostly a stable feature in heritage grammars (Benmanmoun et al., 2013); but see Fenyvesi (2005) who reported some instances of person agreement errors in heritage Hungarian. Polinsky and Scontras (2020) suggested that unlike gender and number, the category of ‘person’ is resilient in heritage grammars because of its inherent indexical nature, that is, the fact that ‘person’ encodes the roles of participant and addressee relative to the speech act (Harley and Ritter, 2002). The present study then indicates that ‘person’ is a resilient feature in heritage grammars even in morphosyntactic contexts where the notionally perceived addressee does not condition verb agreement.

The language questionnaire reported in Table 1 showed that, with the exception of one participant who reported exposure to Spanish at age 11, the target population experienced exposure to Spanish from birth, and they were subsequently exposed to English at approximately age 4. Thus, I assumed that language socialization norms in Spanish were primarily learned at home for most of the participants, a social domain where informal language use is promoted (Dubinina, 2021; He, 2011; Polinsky, 2018). Consequently, RQ2 asked about the social factors that may inform the use of the T/U paradigm in the target heritage population. I predicted that social factors such as age, social rank or the social distance between speaker and addressee would not influence heritage speakers’ use of the *usted* pronoun because these language learners were primarily exposed to informal language practices during heritage language development, and they rarely receive formal education in the heritage language (Carrira and Kagan, 2011; He, 2011; Polinsky, 2018). In fact, participants reported, on average, greater English use at work (86.20%) compared to Spanish (20.71%) (see Table 1).

However, a mixed-effects analysis including all participants (see Table 4) revealed that participants were more likely to use *usted* when addressing female and unknown interlocutors. The reader may recall that proficiency did not reached statistical significance in the analysis including all participants, but this variable reached a significance level in Model-2, which excluded four female participants. This particular finding is perplexing because proficiency was not significant in the female-only analysis either, which included the four female participants excluded in Model-2. Unfortunately, the limited number of male participants (*n* = 5) does not support including participants’ gender as a potential factor in the analysis. Future studies should control for participants’ gender.

Addressee’s gender also emerged as a significant factor in the analysis including all participants (see Table 4), but this factor was more relevant in the female-only analysis (see Table 5). In particular, female speakers used *usted* with unknown male interlocutors but rarely did so with a known male interlocutor (see Figure 1). By contrast, they employed *usted* with both known and unknown female interlocutors. Moreover, while ‘power,’ or perceived social rank, did not strongly affect female participants’ use of *usted* with male addressees, it played a significant role in interactions with female interlocutors (see Figure 2). Taken together, these findings suggest that female speakers were more likely to employ the pronoun that prototypically signals deference in Spanish, namely *usted*, to address female than male addressees, which in turn suggests that female heritage speakers invoked language practices that convey in-group solidarity when interacting with other females. In other words, female heritage speakers may be using *usted* to create social similarity and/or to ensure a female addressee that no imposition is intended (e.g., Brown, 1980).

In the survey data reported in Table 6, for example, participants stated that they would use *usted* with a waitress of similar age because she was providing a service to them, and they wanted to be polite for such disposition. Similarly, they mentioned using *usted* with their grandmothers to show ‘respect.’ Grandparents play a crucial role in heritage language maintenance (Jasso et al., 2024), and the heritage experience more generally (Carrira and Kagan, 2011). Participants’ reference to ‘respect’ may in fact reflect an affective dimension of politeness toward grandparents (see, e.g., Cruz, 2024 on how the diminutive conveys affection toward grandparents in heritage Spanish). In short, both politeness and affection emerged as key pathways through which *usted* indexes in-group solidarity in female-to-female interactions, whereas social distance between speaker and addressee signals deference in female-to-male interactions.

The present study is not the first one to show that Spanish speakers apply the pronoun that prototypically expresses deference to convey in-group solidarity. For instance, Cepeda Ruiz (2017) and Fernández-Mallat and Barrero (2023) found that Colombian men use *usted* to address other men, but they use *tú* to address women. While the use of *usted* in male-to-male dyads is driven by the social connotations attributed to *tú* usage in Colombia (e.g., effeminacy), these studies indicate that *usted* also indexes in-group membership in other speech communities, an indexation that is not necessarily driven by power relations. While the present study supports the well-documented observation that men and women employ different linguistic practices in same-gender groups (Brown, 1980; Lakoff, 1973), future studies should examine how the social conventions governing pronoun choice in the Spanish-speaking world diverge between men and women.

The church is another a space for heritage language socialization, and where politeness is promoted (Joo et al., 2024). For example, in Sigüenza-Ortiz’s (1996) study, heritage bilinguals reported (or were observed) using *usted* at church. Moreover, heritage bilinguals from Tomé, NM and Tucson, AZ reported using *usted* in *compadrazgo* relationships brought into existence through religious ceremonies (Jaramillo, 1988, 1996). As Jaramillo noted, these relationships often involve relatives or close friends, but the transition to a new *compadrazgo* relationship creates special religious and social responsibilities, which in turn promote politeness and mutual respect. Religious celebrations also play a crucial role in the *Quinceañera* tradition among Latina youth in the U. S. (Potowski and Gorman, 2011). In the *Quinceañera* ritual, families engage in *compadrazgo* relationships, and the *Quinceañera* is expected to express gratitude and politeness toward her *padrinos* ‘godparents’ and those who provide financial support for the celebration (Cantú, 2002). Thus, it is fair to suggest that the use of *usted* in these social domains also conveys in-group solidarity, rather than deference rooted in religious ideologies or social hierarchies.

Nevertheless, the use of solidary *usted* in heritage communities does not mean that heritage speakers lack social norms of formal language socialization. For instance, the present study revealed that social distance between speaker and addressee plays a crucial role in informing heritage speakers’ use of formal *usted* in their social interactions. Moreover, and while ‘power’ was not a significant factor in the analysis including all participants, in their self-reported data, all participants reported using *usted* with their professor or potential employer, and the majority did so for the university secretary scenario (95.25%). Finally, in their justifications, participants stated that social rank and professional setting informed their use of *usted*. The majority of the participants (85.71%) reported having taken at least one Spanish language course in college, so it is possible that they learned about these social norms in their language courses in college, although they may not necessarily invoke them in spontaneous speech given their informal language practices in their heritage language(s). Fernández-Mallat and Newman (2021) also found that heritage speakers from New York applied formality with unknown interlocutors, so the present study provides further evidence that social distance is a strong predictor for expressing formality in heritage Spanish.

After completing the VR game, participants selected the pronoun they would use for each of the scenarios they experienced in VR. RQ3 then asked whether participants’ production data would match their self-reported data about the scenarios they experienced in VR. Drawing on Gutiérrez-Rivas (2010) preliminary findings, I predicted higher usage of *usted* in the self-reported data. In Table 6 reporting participants’ perception data, we saw that perceived age and social rank of the addressee influenced participants selection of *usted* for scenarios involving older and socially superior interlocutors. Yet, these factors did not reach statistical significance in the analysis including all participants (see Table 4); and as explained before, ‘power’ reached statistical significance in the female-only analysis for reasons other than expressing deference.

More interestingly, none of the participants mentioned gender as a potential factor informing their use of the *usted* pronoun, but gender was a significant factor across analyses, providing further support for the claim that female speakers used *usted* in female-to-female interactions to convey in-group solidarity and similarity. Finally, participants reported higher usage of *usted* (68.45%) in the survey data compared to their production data (49.40%) for the same target scenarios. In short, participants’ self-reported pronoun usage only partially matched their production data. This finding corroborates Gutiérrez-Rivas’s (2010) preliminary findings that heritage speakers report greater use of formal address compared to their actual use in production. As a reviewer suggests, this particular finding reveals a discrepancy between what speakers believe they do with language and how they actually behave in context. As mentioned before, the majority of existing studies on the social pragmatics of address in heritage Spanish presented self-reported data, limiting their findings to heritage speakers’ perceived use of the T/U paradigm. Future studies should consider eliciting production and perception data from the same participants about the same linguistic variable(s).

While the use of *usted* by female speakers in the present study is somewhat contradictory to the prototypical dimensions of the T/V dichotomy proposed by Brown and Gilman (1960), research shows that bilingualism is a marker of identity for heritage speakers (Carrira and Kagan, 2011; Leeman, 2015; Polinsky, 2018; Valdés, 2005). Consequently, their use of social pragmatics should not be limited to conventional norms in the homeland (Shively, 2021; Pinto, 2018; Polinsky, 2018; Escalante and Quan, 2023). For instance, studies show that heritage bilinguals employ pronouns of address to position themselves as members of a speech community, or to construct social identity (Liebscher et al., 2010; Warditz, 2025). Similarly, they may engage in codeswitching to avoid marking social hierarchies in their discourse (Zentella, 1997). The social pragmatics of address in heritage bilingualism, then, involve a complex set of pragmatic norms sensitive to the heritage experience and bilingual practices. In this sense, the present study provides further evidence for a more contextually situated framework to study address systems cross-linguistically (e.g., Fernández-Mallat and Moyna, 2025). In short, the social pragmatics of address are dynamic and evolving, and they are sensitive to the social norms unique to a speech community.

A note on pragmatic competence is in order. The reader will recall that the experimental design was framed within a study abroad journey to Mexico. In the Introduction section, I noted that speakers must assess the degree to which a particular pronoun of address is rated as an imposition in the target culture. In the literature review, I further reported that Cepeda Ruiz (2018) found that women, compared to men, preferred using the *tú* pronoun in their everyday interactions in present-day Mexico City because this pronoun is more ‘respectful’ and ‘elegant’ than its deferential counterpart. In the present study, however, female heritage speakers were more likely to use *usted* with female interlocutors and, conversely, *tú* with male interlocutors in a study abroad journey to Mexico; and despite the fact that they were presumably exposed to the Mexican variety in their community. In other words, female heritage speakers applied norms of address that are contrary to the host country’s pragmatic norms. This particular finding points to the importance of addressing social pragmatics in heritage language classes (Díaz and Callahan, 2020; Escalante and Quan, 2023).

The setting of the experimental design poses some challenges for broader implications of the results presented here. First, one could argue that the gender effect found in this study could be attributed to the fact that participants were immersed in a foreign country, which could have triggered higher politeness in interactions with Mexican interlocutors. However, Figure 2 showed that the probability of applying formality with male interlocutors is very low (0 to 2%) compared to the probability of employing formality with female interlocutors (79 to 38%). Thus, context *per se* cannot explain the gender effect found in this study. Nevertheless, the results presented here may not necessarily reflect heritage speakers’ social pragmatics of address in interactions with other members of their speech community in the U. S.-context. Similarly, virtual reality does not replace real-world interactions in their totality. Future studies are needed to understand heritage speakers’ social pragmatics of address among members of their speech community. I hope that the experimental design presented here, which includes the VR game available in the author’s OSF repository, may inspire other researchers to replicate this study with other heritage communities in the U. S.-context.

Moreover, future studies should consider examining heritage speakers along the proficiency continuum and analyze the role of proficiency and formal instruction in promoting heritage speakers’ knowledge of social pragmatics. A larger number of scenarios involving more social relations may increase statistical power to tease out the potential role of social factors such as age and power in heritage language socialization. Finally, as a reviewer points out, future studies should include cognitive functioning measures to control for individual differences, especially because the immersive nature of VR may increase cognitive load. Despite the limitations of the present study, virtual reality is a promising technology for studying addressee-dependent linguistic variables (see Wirtz et al., 2024 for application of VR in second language acquisition research). As this technology continues to evolve, researchers could explore ways to benefit from this technology for linguistics research, especially in the interplay between different modalities in dynamic and communicative real-world contexts.

## 6 Conclusion

This study employed virtual reality to examine the social dimensions that may inform Spanish heritage speakers’ use of pronouns of address, as well as the extent to which the feature geometry of these pronouns constrain their use in spontaneous speech. Unlike survey questionnaires, virtual reality gives the speaker/participant the opportunity to assess social relations between speaker and addressee in real time. The study controlled for the interlocutor/addressee’s perceived age, gender, and social rank, as well as the social distance relation between speaker and interlocutor. While the results for the most part corroborate previous studies in term of the social factors that inform heritage speakers’ pronoun usage in heritage communities in the U.S. (e.g., the role of social distance), the analysis revealed a gender effect that is novel to the literature. Specifically, female participants were more likely to employ formality (e.g., use of the *usted* pronoun) when addressing female rather than male interlocutors. I interpreted this finding to indicate that female participants applied language practices that convey in-group solidarity and social similarity, at least in a study abroad context. Moreover, the study revealed a discrepancy between what speakers believe they do with language and how they actually behave in context, highlighting the importance of eliciting production and perception data from the same participants about the same linguistic variable(s). By studying the nuances of address systems in virtual worlds, the present study contributes to our current understanding of pragmatics in heritage bilingualism.

## Data Availability

The datasets presented in this study can be found in online repositories. The names of the repository/repositories and accession number(s) can be found at: Open Science Framework: https://osf.io/trcuh/.

## Footnotes

1 Instead, some extra syntactic element is required for third-person singular verb agreement with *usted* (see Collins and Ordóñez, 2021 for a possible analysis). Similarly, the plural counterpart of *usted* also requires activation of the ‘addressee’ node dependent on PARTICIPANT in (1), but it exhibits third-person plural verb agreement (e.g., *Ustedes tienen*); in fact, *ustedes* is the only form for 2nd person plural reference in non-Peninsular varieties.

2 This discourse-driven morphosyntactic mismatch is common across languages that express formality. For example, in Italian, second-person singular formal *Lei* is third-person singular and feminine but refers to a single addressee of either gender; similarly, in German, second-person singular formal *Sie* is third-person plural feminine but refers to a single addressee of either gender (Portner et al., 2019).

3
https://osf.io/trcuh/

4 We created all the scenarios employed in the present study from scratch. The number of scenarios was limited to eight because of the limited resources allocated to this project.

5 Timing of the virtual experience was carefully tested out with multiple participants to determine the timing of the expected response. Participants were told that the game was timed and that they might be cut off during their interaction.
