# Supplementary material for: The social pragmatics of address in heritage Spanish: a virtual reality study
Source: Front Psychol. 2026 Feb 24;17:1719331. doi: 10.3389/fpsyg.2026.1719331 (PMC12971648; doi:10.3389/fpsyg.2026.1719331)
Supplement: Supplementary file 1 [file Data_Sheet_1.pdf]

Supplementary Material

Table S1 provides a summary of the scenarios used in the virtual reality game. In these scenarios, social factors are controlled from the addressee's perspective.

Table S1. Characteristics of the simulated scenarios

Virtual Scenario	Age of agent	Gender of agent	Power relation	Distance
V1: Requesting phone at airport from stranger	Same as participant	Male	Absent, stranger-foreigner interaction	Unknown
V2: Requesting taxi ride to university	Older than participant	Male	Absent, taxi driver-foreigner interaction	Unknown
V3: Requesting info. from university secretary at office	Older than participant	Female	Present, university secretary-foreign student interaction	Unknown
V4: Ordering food at college cafeteria	Same as participant	Female	Absent, waiter-foreigner interaction	Unknown
V5: Requesting notes from classmate	Same as participant	Male	Absent, peer-peer interaction	Known
V6: Requesting make-up exam from professor at office	Older than participant	Female	Present, foreign student-professor interaction	Known
V7: Interview with potential employer at in office setting	Older than participant	Male	Present, foreign student-potential employer interaction	Unknown
V8: Invitation to grandmom to visit Mexico for graduation	Older than participant	Female	Present, older family member-grandchild interaction	Known

Table S2 provides the full results of the immersiveness questionnaire that participants took after completing the virtual reality game. "V1" stands for "scenario 1," and so on.

Table S2. Participants' ratings of immersiveness by scenario

Scenario	Mean (SD)	Min–Max
V1	3.66 (1.11)	1–5
V2	3.74 (1.16)	1–5
V3	3.76 (0.98)	2–5
V4	4.11 (1.01)	2–5
V5	3.78 (0.97)	2 –5
V6	4.14 (0.89)	2–5
V7	3.33 (1.35)	1–5
V8	4.23 (1.14)	1–5

Notes: Immersiveness Questionnaire scale 1= not at all immersed, 5=very immersed

Table S3 provides the results for the model without the three participants who defaulted to *tú* pronoun usage and the one participant who reported exposure to Spanish at age 11 (Model-2 in Section 3.5 of the article).

Table S3. Participants (n=38) likelihood of expressing formality through the *usted* pronoun

Parameters	Estimates	*SE*	95% CI	*z*	*p*
(Intercept)	1.11	0.64	[−0.145, 1.88]	2.37	0.083
$\text{Gender}_{\text{male vs. female}}$	−2.98	0.93	[−4.81, −1.15]	−3.20	0.001**
$\text{Distance}_{\text{known vs. unknown}}$	−3.06	0.88	[−4.80, −1.34]	−3.47	0.000***
$\text{Power}_{\text{present vs. absent}}$	0.73	0.43	[−0.10, 1.58]	1.71	0.087
$\text{Proficiency}_{\text{Spanish}}$	0.92	0.43	[0.09, 1.77]	2.17	0.029
**Random Effects**	**Variance**	**SD**			
Intercept | subject	7.61	2.75			
Intercept | scenario	0.79	0.88			

Note. The 95% CIs were approximated using the Wald method; *p < 0.05; **p < 0.01; ***p < 0.001.

Table S4 provides a summary of the words and phrases that participants wrote in the Language Awareness Questionnaire to justify their pronoun selection for the target scenarios.

Table S4. Participants' justifications for selecting either *tú* or *usted* in the target scenarios

	**Explanation for TU selection**	**Explanation for USTED selection**
V1	Similar in age	unknown person
	informal setting	politeness/respect
	requesting service	asking for a favor
	equal power	
V2	equal power	older person
	informal setting	politeness/respect
	unknown person	unknown person
		asking for a favor
V3	informal setting	older person
	female interlocutor	power/title
		politeness/respect
		providing service
		professional setting
V4	similar age	politeness/respect
	informal setting	providing service/work setting
		unknown person
V5	similar age	
	equal/peer/friend	
	informal setting	
V6		older person
		power/title
		politeness/respect
		formal setting
		formal request/favor
V7		older person
		power/title
		professional setting
		unknown person
		politeness/respect
		desirable job
V8	family/known person	upmost respect
	informal setting	elder family member
	direct request for informality	
